# Interstitial Telomeric Motifs in Squamate Reptiles: When the Exceptions Outnumber the Rule

**DOI:** 10.1371/journal.pone.0134985

**Published:** 2015-08-07

**Authors:** Michail Rovatsos, Lukáš Kratochvíl, Marie Altmanová, Martina Johnson Pokorná

**Affiliations:** 1 Department of Ecology, Faculty of Science, Charles University in Prague, Prague, 128 44, Czech Republic; 2 Institute of Animal Physiology and Genetics, The Academy of Sciences of the Czech Republic, Liběchov, 277 21, Czech Republic; University of Florence, ITALY

## Abstract

Telomeres are nucleoprotein complexes protecting the physical ends of linear eukaryotic chromosomes and therefore helping to ensure their stability and integrity. Additionally, telomeric sequences can be localized in non-terminal regions of chromosomes, forming so-called interstitial telomeric sequences (ITSs). ITSs are traditionally considered to be relics of chromosomal rearrangements and thus very informative in the reconstruction of the evolutionary history of karyotype formation. We examined the distribution of the telomeric motifs (TTAGGG)_n_ using fluorescence *in situ* hybridization (FISH) in 30 species, representing 17 families of squamate reptiles, and compared them with the collected data from another 38 species from literature. Out of the 68 squamate species analyzed, 35 possess ITSs in pericentromeric regions, centromeric regions and/or within chromosome arms. We conclude that the occurrence of ITSs is rather common in squamates, despite their generally conserved karyotypes, suggesting frequent and independent cryptic chromosomal rearrangements in this vertebrate group.

## Introduction

Telomeres are nucleoprotein complexes that protect the physical ends of linear eukaryotic chromosomes, playing a crucial role in maintaining chromosome stability and integrity [1]. In all vertebrates the DNA component of telomeres consists of the non-coding (TTAGGG)_n_ motif [2], which produce long tandem repetitions varying greatly in size between species, individuals and even cell types [3]. The telomeric motif demonstrates a remarkable evolutionary conservation across vertebrate species [4,5]. The telomere-specific complex associated with the telomeric sequence has been described as "shelterin" [6]. Shelterin is composed of three proteins (TRF1, TRF2 and POT1) that directly recognize the (TTAGGG)_n_ motif, and are interconnected by three additional proteins (TIN2, TPP1 and Rap1) to form a duplex structure [7] (for a review see [3]). The telomeric motif is synthesized by telomerase, a reverse transcriptase-like enzyme, which contains an RNA subunit and a catalytic protein subunit called telomerase reverse transcriptase [8]. Telomerase uses the RNA template to add additional sequences directly to the telomeres [9]. In humans telomerase is expressed in embryonic tissues and specific germline cells whilst in adults, the enzyme can be detected mainly in the testis, and is absent in most normal somatic cells, in non-dividing oocytes and mature spermatozoa [10,11].

The main role of telomeres is to protect the edges of the linear chromosomes from degradation, recombination or fusion, preventing the chromosomal ends from being recognized as double-strand brakes by DNA repair machinery [3]. Furthermore, the DNA replication machinery cannot completely replicate the ends of linear chromosomes as there would not be any template strands to guide its synthesis ("end replication problem") [12]. In each cell division 50–200 bp are erased from the edges of the chromosomes decreasing the chromosome length and eventually affecting the inner genetic loci. Telomerase preserves the edge of the chromosomes by adding "expendable" telomeric motifs *de novo* [3]. However, in several cell types, such as human somatic cells, telomeres become shorter after subsequent replications [13], resulting in a minimum amount of telomeric sequence, leading to replicative senescence and ultimately cell death [14]. This phenomenon has been described as the "telomere hypothesis of cellular aging", a theory that proposes that telomeres serve as a "mitotic clock" controlling lifespan [15].

An additional role of telomeres is the maintenance of the chromosome topology in the nucleus matrix and the correct alignment of chromosomes for recombination during the first meiotic prophase [16–18]. Another important function of telomeres is the silencing of adjacent genes, a phenomenon known as "telomere position effect" [19,20].

As well as the crucial role of telomeres at the edges of chromosomes, non-terminal telomeric motifs known as interstitial telomeric sequences (ITSs) [21] or interstitial telomeric repeats (ITRs) [3], have been observed in many species. The pioneer publication by [5] provided the first cytogenetic evidence of this, reporting that 55 out of the 100 studied species of vertebrates had ITSs. Many more cases were described in the following years in vertebrates, including amphibians [22], fish [23], birds [24], rodents [25–27], marsupials [28–30] and primates [31].

Based on sequence organization and genomic location, Ruiz-Herrera et al. [21] identified two different types of ITSs: short ITSs (s-ITSs) and heterochromatic ITSs (het-ITSs). Other authors have classified ITSs in more detailed categories as short ITSs, long subtelomeric ITSs, fusion ITSs and pericentromeric ITSs [32]. S-ITSs are short sized telomeric repetitions located in internal sites of chromosomes, present in all completely sequenced mammalian genomes (at least 83 in human, 79 in chimpanzee, 244 in mouse and 250 in rat), but often not detectable by cytogenetic techniques such as fluorescent *in situ* hybridization (FISH) [21]. It was initially thought that s-ITSs were derived from the telomeric fusion of ancestral chromosomes [33]. However, recent studies concluded that s-ITSs are not in fact associated with chromosomal rearrangements [34] but instead were probably inserted by telomerase during the repair of DNA double-strand breaks [21,35,36]. This hypothesis is supported by the frequent association of transposable elements such as SINEs and LINEs with s-ITSs [37].

Het-ITSs are large stretches of telomeric sequences (up to hundreds of kb) localized mainly in heterochromatic chromosomal regions such as in centromeric or pericentromeric areas or within the chromosome arms. In contrast to s-ITSs, het-ITSs are only present in a limited number of species and it is widely believed that they correspond to the remnants of ancestral chromosomal rearrangements which occurred during karyotype evolution [38,39].

As far as we know, ITSs have been described in only 22 lizard species and never in snakes [5,40–53]. In general, squamate reptiles are often considered as a group with evolutionary conserved karyotypes. This view has been supported by classical cytogenetics techniques such as conventional staining and C-banding (e.g. [54–56]) as well as by chromosome painting [57–60], gene mapping [45,61,62], and qPCR mapping of genes linked to sex chromosomes [63–66].

Considering that particular types of ITSs represent relics of chromosome rearrangements, the conservation of karyotypes in squamates suggests that ITSs should be relatively rare in this group. In order to test this hypothesis, we reviewed published data on the occurrence of ITSs and supplemented it with our novel description of ITSs distribution in 13 species of squamates based on FISH experiments.

## Material and Methods

### Specimens and chromosomal preparations

**Fig 1:**
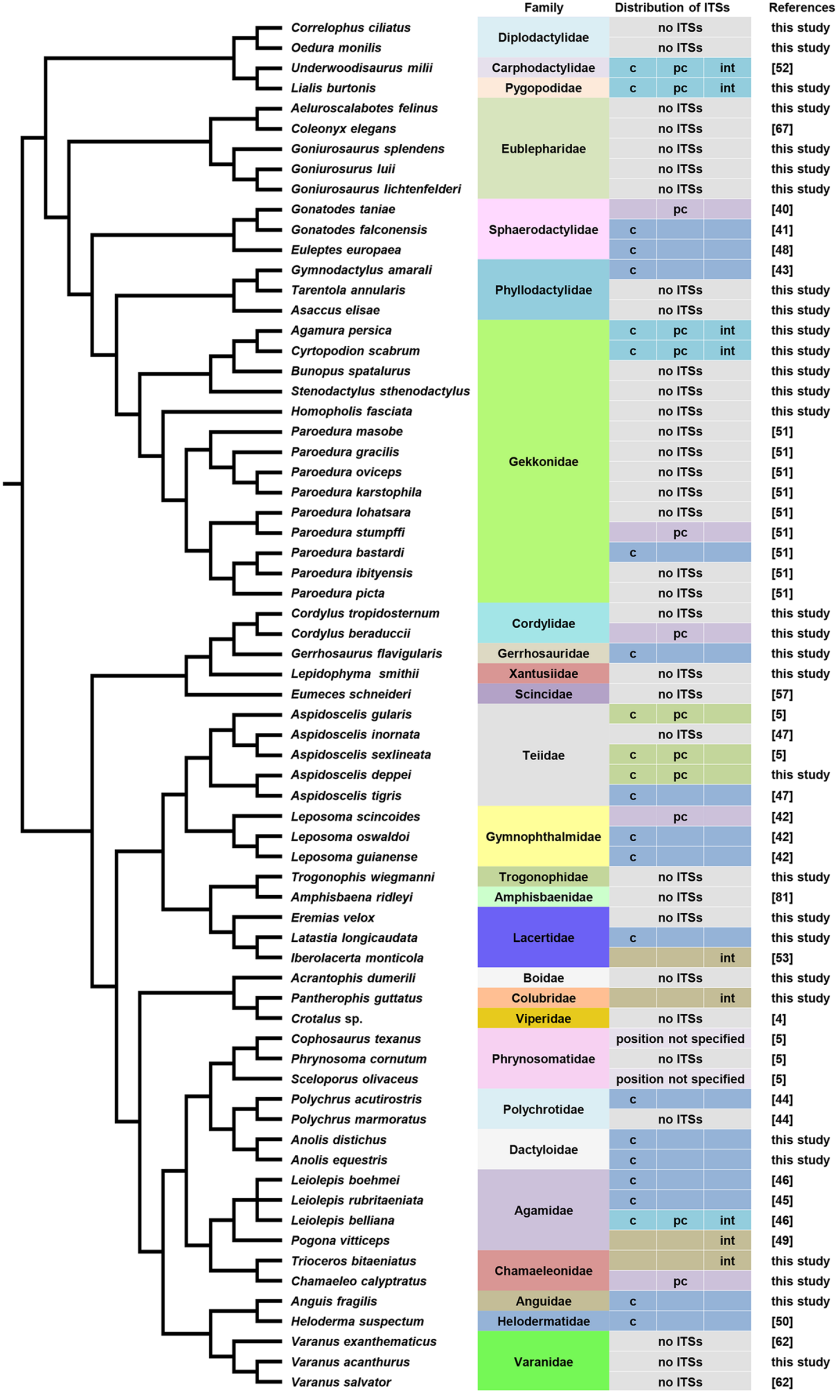
The position of telomeric sequences in 68 species of lizards and snakes. The telomeric motif (TTAGGG)_n_ was detected in the normal, terminal position of all chromosomes. In addition to the terminal topology, interstitial telomeric repeats (ITSs) were detected in several species within centromeric (c), pericentromeric (pc) and/or intermediate (int) chromosomal regions.

The distribution pattern of telomeric motifs was studied in the karyotypes of 30 species of squamate reptiles (28 lizards and 2 snakes), belonging to 17 families (Fig 1) from our collection of metaphase chromosome spreads. The specimens originated from pet trade (the companies Animalfarm CZ, Zoopet Sandy, Happy Reptiles, B.A.R. and Zoo Shop Želvička) and were maintained in the reptile breeding laboratory of the Faculty of Science, Charles University in Prague, Czech Republic (accreditation No. 24773/2008-10001). Blood samples were taken from caudal or brachial vessels. The animal procedures were carried out under the supervision and with the approval of the Ethics Committee of the Faculty of Science, Charles University in Prague followed by the Committee for Animal Welfare of the Ministry of Agriculture of the Czech Republic (permission No. 29555/2006-30). Metaphase chromosome spreads were prepared from whole blood cell cultures following the previously described protocol [67] with slight modifications. Briefly, the small amount (approx. 40 μl) of the peripheral blood was cultured for a week at 30°C in Dulbecco’s Modified Eagle’s Medium (Sigma-Aldrich), enriched with 10% fetal bovine serum (Baria), 0.5% penicillin/streptomycin solution (Gibco), 1% L-glutamine (Sigma-Aldrich), 3% phytohaemagglutinin (Gibco), and 1% lipopolysaccharide (Sigma-Aldrich). Chromosome preparations were made following standard procedures including a 3.5 hours colcemid treatment, hypotonization, and fixation in 3: 1 methanol:acetic acid.

### Fluorescent in situ hybridization (FISH)

A specific probe for the telomeric motif (TTAGGG)_n_ was produced and labelled with dUTP-biotin by PCR, using the primers (TTAGGG)_5_ and (CCCTAA)_5_, without a DNA template, according to the methodology of Ijdo et al. [68]. Briefly, a PCR reaction was performed in 50 μl final volume, including 0.4 μl of each primer (5 pmol/μl), 5 μl of 10× PCR buffer (Bioline), 2.5 μl MgCl2 (50mM), 1 μl dATP, dCTP, dGTP (10 mM each), 0.7 μl dTTP (10 mM), 1 μl dUTP-biotin (1 mM) and 1 μl BioTaq DNA polymerase (5 U/μl, Bioline). The PCR cycling conditions were as follows: 5 min at 94°C, 10 cycles of 1 min at 94°C, 30 sec at 55°C and 1 min at 72°C, followed by 30 cycles of 1 min at 94°C, 30 sec at 60°C and 30 sec at 72°C, with a final step of 5 min at 72°C. The PCR product was precipitated and re-suspended in 300 μl of hybridization buffer (50% formamide/2× SSC).

Prior to *in situ* hybridization, 10 μl of the telomeric probe per slide was denatured at 75°C for 10 min and then chilled on ice for 10 min. In parallel, the metaphase slides were subsequently treated with RNase, pepsin, fixed with 4% formaldehyde, dehydrated through a series of 70%, 85% and 100% ethanol washes, denatured in 70% formamide/2× SSC at 75°C for 4 min, dehydrated again and air dried. Afterwards, the probe was applied to each slide and incubated at 37°C for 16–24 hours.

Post-hybridization washes were subsequently carried out in 50% formamide/2× SSC at 42°C (3 × 5 min) and in 2× SSC (2 × 5 min). The slides were incubated in 100 μl of 4× SSC/5% blocking reagent (RocheAρχήφόρμαςTέλοςφόρμας) at 37°C for 45 min. The telomeric signal was detected using a modified avidin-FITC/biotinylated anti-avidin protocol for FITC signal amplification. In detail, we prepared two different solutions: a primary antibody solution with 300 μl of 4× SSC/5% blocking reagent, including 0.3 μl avidin-FITC per slide (Vector laboratories) and a secondary antibody solution with 200 μl of 4× SSC/5% blocking reagent, including 2μl biotinylated anti-avidin per slide (Vector laboratories). The FITC signal was enhanced by five subsequent applications of the primary (three times) and the secondary (two times) antibody solutions at 37°C for 30 min each, using 100 μl of each antibody solution per slide, with intermediate washes in 4× SSC/0.05% Tween20 (3 × 5 min). Afterwards, the slides were dehydrated through an ethanol series, air dried, counterstained with 4',6-diamidino-2-phenylindole (DAPI) and mounted with Vectashield anti-fade medium (Vector Laboratories).

### Microscopy and image analyses

An Olympus Provis AX70 fluorescence microscope with a DP30BW digital camera was used to take grayscale images that were processed with DP manager imaging software (Olympus) to record the pattern of the telomeric repeats within the chromosomal metaphases.

### Phylogenetic distribution

The phylogenetic distribution of the presence/absence of ITSs across squamate reptiles was visualized using Mesquite v.2.75 [69], based on the phylogenetic tree topology of Pyron et al. [70].

## Results

FISH with telomeric probe proved to be a valuable tool in revealing the topology of the telomeric motif (TTAGGG)_n_ in the karyotypes of squamate reptiles. Based on the distribution and the putative origin of the telomeric sequences within the chromosomes we distinguished the following topologies in the karyotypes:

### Karyotypes with only terminal distribution of telomeres

**Fig 2:**
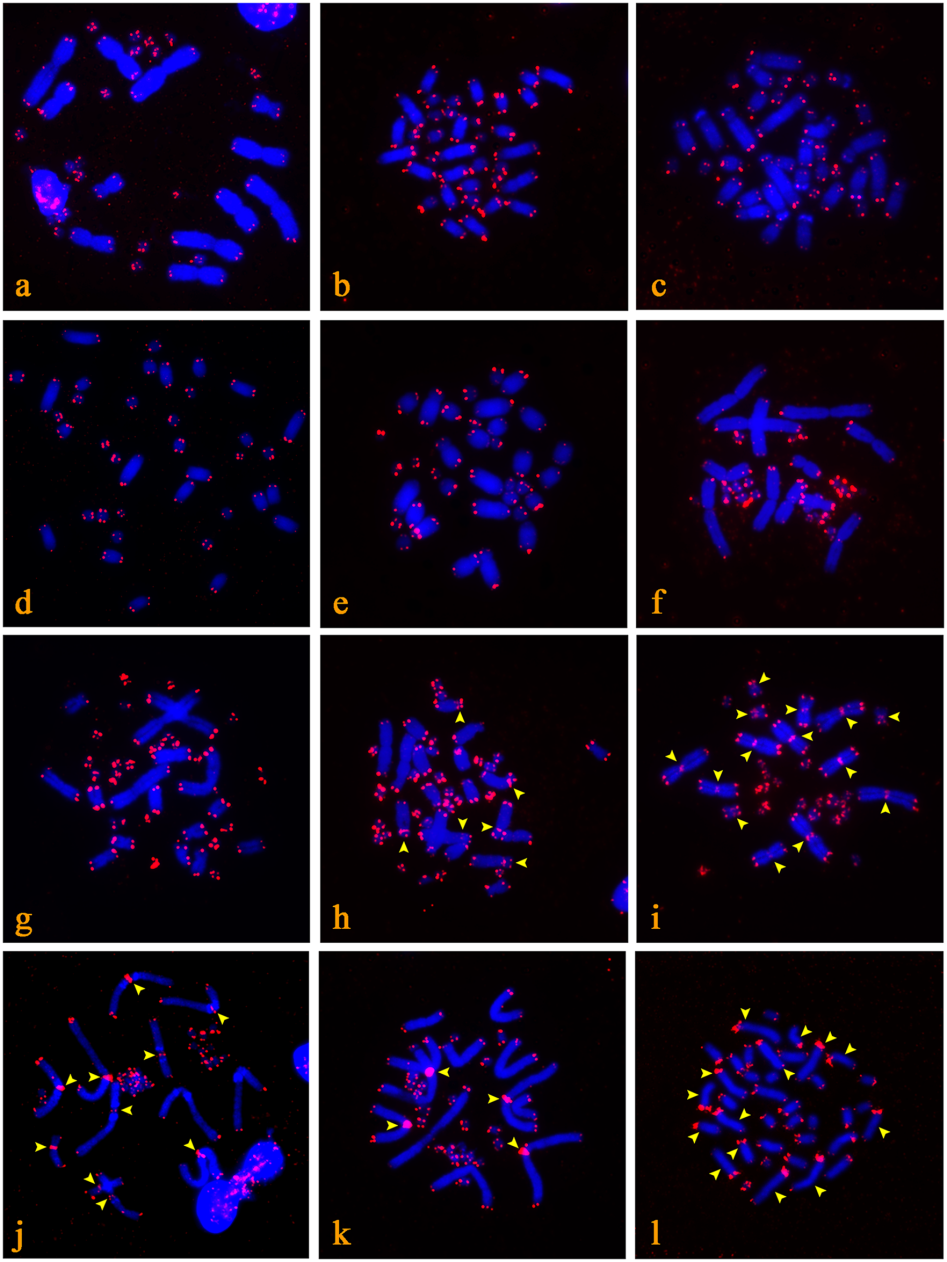
The position of the telomeric sequences in the chromosomal spreads, as revealed by FISH. Exclusively terminal distribution: *Cordylus tropidosternum* (a), *Correlophus ciliatus* (b), *Oedura monilis* (c), *Goniurosaurus luii* (d), *Homopholis fasciata* (e), *Trogonophis wiegmanni* (f), *Varanus acanthurus* (g). ITSs in centromeric regions: *Anguis fragilis* (h), *Anolis distichus* (i), *Anolis equestris* (j), *Gerrhosaurus flavigularis* (k), *Latastia longicaudata* (l). Chromosomes are stained with DAPI (blue), while the FITC signal from the telomeric probe is red. Chromosomes with ITSs are indicated by arrows.

In 17 species we observed telomeric sequences only at the expected terminal positions at the ends of the chromosomes. Specifically, this group includes the species *Acrantophis dumerili* (Boidae), *Cordylus tropidosternum* (Fig 2a) (Cordylidae), *Correlophus ciliatus* (Fig 2b), *Oedura monilis* (Fig 2c) (Diplodactylidae), *Aeluroscalabotes felinus*, *Goniurosaurus lichtenfelderi*, *G*. *luii* (Fig 2d), *G*. *splendens* (Eublepharidae), *Bunopus spatalurus*, *Homopholis fasciata* (Fig 2e), *Stenodactylus sthenodactylus* (Gekkonidae), *Eremias velox* (Lacertidae), *Asaccus elisae*, *Tarentola annularis* (Phyllodactylidae), *Trogonophis wiegmanni* (Fig 2f) (Trogonophidae), *Varanus acanthurus* (Fig 2g) (Varanidae) and *Lepidophyma smithii* (Xantusiidae).

### Karyotypes with ITSs in centromeric regions

Five species had karyotypes with telomeric motifs at the terminal positions of all chromosomes, and additional ITSs in centromeric regions of one or more chromosomal pairs. In detail, ITSs were detected at the centromeres of three submetacentric pairs in *Anguis fragilis* (Fig 2h) (Anguidae), seven chromosomal pairs in *Anolis distichus* (Fig 2i), five chromosomal pairs in *Anolis equestris* (Fig 2j) (Dactyloidae), two large metacentric pairs in *Gerrhosaurus flavigularis* (Fig 2k) (Gerrhosauridae) and five chromosomal pairs in *Latastia longicaudata* (Fig 2l) (Lacertidae).

### Karyotypes with ITSs in pericentromeric regions

**Fig 3:**
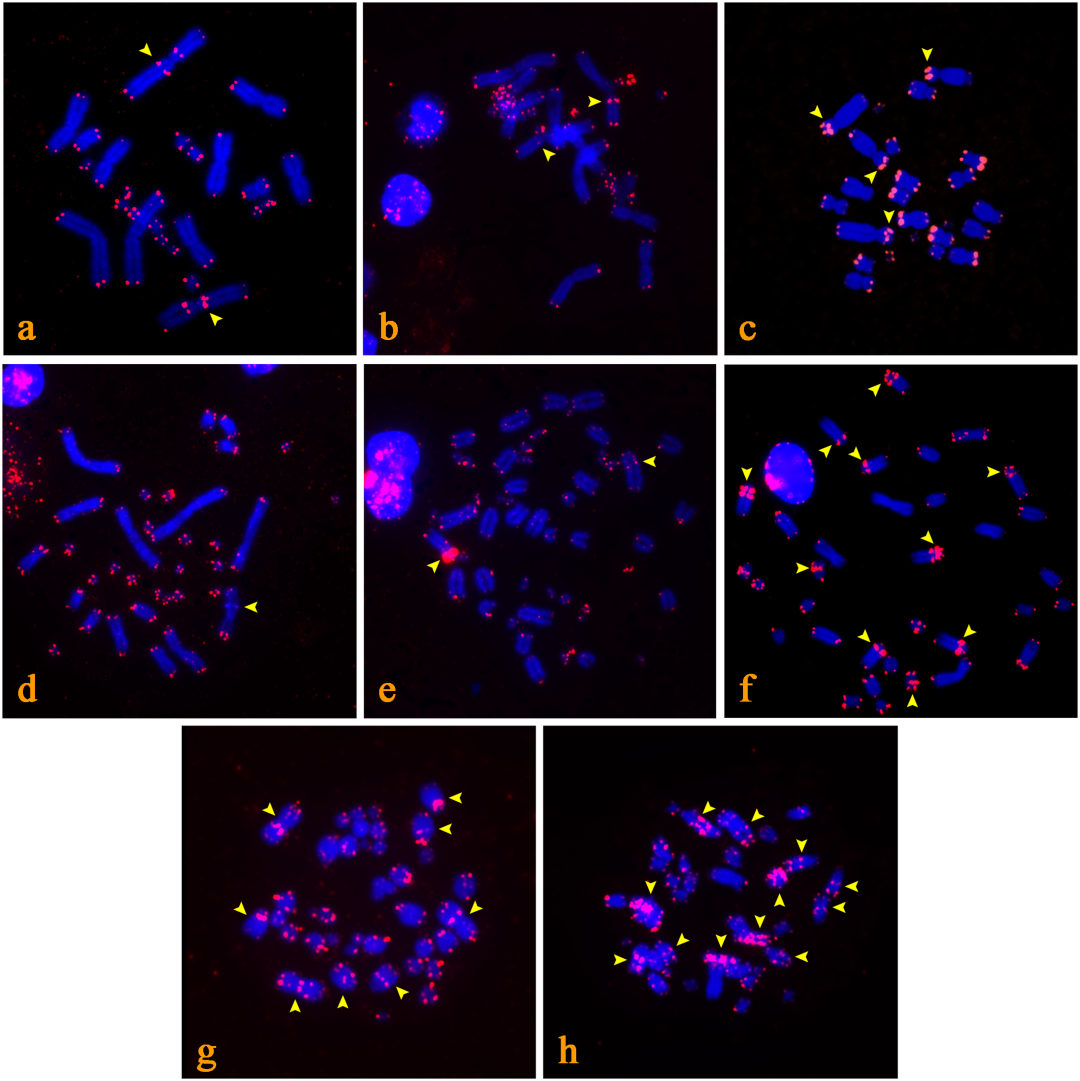
The position of the telomeric sequences in the chromosomal spreads, as revealed by FISH. ITSs in pericentromeric regions: *Chamaeleo calyptratus* (a), *Cordylus beraduccii* (b). ITSs in intermediate positions: *Trioceros bitaeniatus* (c), *Pantherophis guttatus* (d). ITSs in numerous positions: *Aspidoscelis deppei* (e), *Lialis burtonis* (f), *Cyrtopodion scabrum* (g), *Agamura persica* (h). Chromosomes are stained with DAPI (blue), while the FITC signal from the telomeric probe is red. Chromosomes with ITSs are indicated by arrows.

Two lizard species, *Chamaeleo calyptratus* (Fig 3a) (Chamaeleonidae) and *Cordylus beraduccii* (Fig 3b) (Cordylidae), had telomeric motifs at the terminal positions of all chromosomes, and additional ITSs in pericentromeric regions of the largest metacentric chromosome pair.

### Karyotypes with ITSs within chromosome arms

Two species exhibit ITSs signals between terminal telomeres and the centromeric/pericentromeric regions. In *Trioceros bitaeniatus* (Fig 3c) (Chamaeleonidae), ITSs are present at intermediate positions on six chromosomal pairs, and in *Pantherophis guttatus* (Fig 3d) (Colubridae) there is an interstitial telomeric band on a medium sized metacentric chromosome.

### Karyotypes with ITSs in numerous positions

Finally, in four species we observed ITSs in numerous positions on chromosomes including all of the above mentioned categories (centromeric, pericentromeric and within the chromosome arms). This extensive accumulation was observed in *Aspidoscelis deppei* (Fig 3e) (Teiidae), *Lialis burtonis* (Fig 3f) (Pygopodidae), *Cyrtopodion scabrum* (Fig 3g) and *Agamura persica* (Fig 3h) (Gekkonidae).

The phylogenetic distribution of ITSs (Fig 1) suggests that in general ITS emergence/loss is evolutionary dynamic across squamates. A high incidence of ITSs is present in the sister families Teiidae and Gymnophthalmidae, while Iguania also possess the tendency to accumulate ITSs in their genomes, yet ITSs appear to have a rather random distribution across the other squamate lineages.

## Discussion

ITSs are present in members of all major lineages of squamates (Fig 1). Taking into account previous publications and our results, only 48.5% of squamates (n = 33 species) demonstrate the normal, expected distribution of telomeres at the edges of all chromosomes. Surprisingly, around half of the studied squamate species (51.5%, n = 35) show ITSs in centromeric regions, pericentromeric regions and/or within chromosomal arms. It therefore appears that the existence of ITSs in squamate genomes is not an exception, but rather a common event (Fig 1). The species with karyotypes demonstrating ITSs seem to be more or less randomly distributed across the phylogeny of squamates, with several families including species with both normal terminal telomeres and ITSs, while a higher incidence of ITSs typifies sister families Teiidae and Gymnophthalmidae and the lineage Iguania (Fig 1). Nevertheless, it should be noted that although our sampling includes members of most major lineages of Squamata, the total number of species tested for the presence of ITSs is still quite small and somewhat patchy, which precludes detailed statistical analyses of ITSs correlations and phylogenetic distribution.

ITSs are commonly observed in centromeric regions of both bi-armed and acrocentric chromosomes. ITSs in the centromeres of bi-armed chromosomes might have originated from the remains of “old” terminal telomeres after Robertsonian fusion (e.g. in the gecko *Gymnodactylus amarali*; [43]), while the ITSs on the centromeres of acrocentrics may be the result of extensive amplification due to their proximity to satellite sequences. It is well-documented that telomeres can be part of centromere repetitive elements [26,71]. In a recent study [71], it was demonstrated that all centromeres of a vole species exhibit co-localization of ITSs with three other satellite sequences. These ITSs were cloned and sequenced, demonstrating 87% to 94% similarity to the terminal telomeric motif (TTAGGG)_n_ [26]. The extensive amplification of ITSs in the centromeres of acrocentric chromosomes covering a large part of the centromeric region can be observed in several squamates, such as *Latastia longicaudata* (Fig 2l). However, further studies are needed to reveal the type of repetitive elements co-localized with ITSs in squamates and to show if species with ITSs share a similar content of satellite sequences within their centromeres.

Extended ITSs in intermediate positions in several lizard species may reflect remnants of past intrachromosomal rearrangements. Although sauropsids possess a relatively low rate of interchromosomal rearrangements, it has been shown in birds that intrachromosomal rearrangements occur rather frequently [72–74]. In fact, whereas no interchromosomal rearrangements have been documented in the microchromosomes of the chicken, turkey or zebra finch, there have been numerous intrachromosomal rearrangements recorded in these species [74]. In the same context, several pericentromeric inversions have been discovered in the chromosomal pairs 1–4 of *Anolis carolinensis* using *in situ* hybridization with BACs [75]. Furthermore, the comparison between the homologous part of chromosome 15 of the chicken and chromosome X of *Anolis carolinensis* revealed extensive synteny of the gene content, and numerous intrachromosomal, but few interchromosomal rearrangements in the studied chromosomal region [66]. Evidence for numerous intrachromosomal, but rare interchromosomal rearrangements based on interspecific chromosome painting was recently presented in geckos [76].

In some cases telomeric-like sequences appear to accumulate at the heterochromatic part of sex chromosomes. The exact role of the accumulation of ITSs and satellite sequences (for a review see [77]) on the highly heterochromatic W (e.g. in the gecko *Underwoodisaurus milii*; [52]) or Y chromosomes remains unclear. It has been speculated that the accumulation of repetitive sequences on one pair facilitates the suppression of recombination between sex chromosome homologues, enabling the accumulation of sexually beneficial mutations on respective sex chromosomes. Some authors however suggest that the repetitive sequences may accumulate near the sex determining locus as a result of the suppression of recombination rather than inducing it ([77] and references within).

Finally, closely related species with similar chromosome morphology seem to possess different patterns of ITSs distribution, e.g. species of the genus *Anolis*, *Cordylus*, *Paroedura* [51] and *Leiolepis* [45,46] (Fig 1). Such differences could be explained either by the dynamic nature of ITSs (e.g. as part of satellite DNA or transposable elements) or cryptic rearrangements. Many reptile linages, with the exception of their avian clade, show persistent telomerase activity in the somatic tissues of adults which might not only explain their extensive tissue regeneration potential [78], but also the existence of ITSs accumulation at numerous positions in their genome (Fig 1). In fact, telomerase appears to be active in all of the tissues of adult *Aspidoscelis sexlineata* [78], a species with ITSs accumulation (Fig 1; [5]). Furthermore, skin fibroblasts from the blue racer snake (*Coluber constrictor*) show increased telomerase activity after a high number of generations *in vitro* [79]. Moreover, telomere length does not decrease with age in the water python (*Liasis fuscus*), but instead increases from approximately 7 kb at hatching to 28 kb at adult age [80], providing another exception to the hypothesis of “cellular aging”.

In summary, we detected ITSs for the first time in the genomes of 13 species of squamate reptiles and documented that ITSs were observed in approximately half (35 out of 68) of the species of lizards, snakes and amphisbaenians, e.g. [81], studied so far. Therefore we can conclude that the occurrence of ITSs is surprisingly high in this group of vertebrates which has otherwise stable and conserved karyotypes. This discrepancy suggests that, similar to birds, squamate reptiles may have a rather high rate of intrachromosomal rearrangement and a low rate of interchromosomal rearrangement. The origin of ITSs in some species of squamate reptiles may however be attributed to other factors such as high telomerase activity and/or the repair mechanisms of double-strand breaks, e.g. triggered by the activity of transposable elements. Future studies should be devoted to increasing the taxonomic scope of the testing of ITSs distribution across squamates and to address questions regarding the functional importance of these unusually frequent elements in squamate genomes.

## References

[1] Blackburn EH, Greider CW (1995) Telomeres. Cold Spring Harbor Laboratory Press.

[2] Moyzis RK, Buckingham JM, Cram LS, Dani M, Deaven LL, Jones MD, et al (1988) A highly conserved repetitive DNA sequence, (TTAGGG)_n_, present at the telomeres of human chromosomes. Proc Natl Acad Sci U S A 85:6622–6626. 3413114 10.1073/pnas.85.18.6622 PMC282029

[3] Bolzán AD, Bianchi MS (2006) Telomeres, interstitial telomeric repeat sequences, and chromosomal aberrations. Mutat Res 612:189–214. 16490380 10.1016/j.mrrev.2005.12.003

[4] Meyne J, Ratliff RL, Moyzis RK (1989) Conservation of the human telomere sequence (TTAGGG)_n_ among vertebrates. Proc Natl Acad Sci U S A 86:7049–7053. 2780561 10.1073/pnas.86.18.7049 PMC297991

[5] Meyne J, Baker RJ, Hobart HH, Hsu TC, Ryder OA, Ward OG, et al (1990) Distribution of non-telomeric sites of the (TTAGGG)_n_ telomeric sequence in vertebrate chromosomes. Chromosoma 99:3–10. 2340757 10.1007/BF01737283

[6] de Lange T (2005) Shelterin: the protein complex that shapes and safeguards human telomeres. Genes Dev 19:2100–2110. 16166375 10.1101/gad.1346005

[7] Griffith JD, Comeau L, Rosenfield S, Stansel RM, Bianchi A, Moss H, et al (1999) Mammalian telomeres end in a large duplex loop. Cell 97:503–514. 10338214 10.1016/s0092-8674(00)80760-6

[8] Greider CW, Blackburn EH (1985) Identification of a specific telomere terminal transferase activity in *Tetrahymena* extracts. Cell 43:405–413. 3907856 10.1016/0092-8674(85)90170-9

[9] Blackburn EH (2005) Telomeres and telomerase: their mechanisms of action and the effects of altering their functions. FEBS Lett 579:859–862. 15680963 10.1016/j.febslet.2004.11.036

[10] Nakamura TM, Morin GB, Chapman KB, Weinrich SL, Andrews WH, Lingner J, et al (1997) Telomerase catalytic subunit homologs from fission yeast and humans. Science 277:955–959. 9252327 10.1126/science.277.5328.955

[11] Liu L, Bailey SM, Okuka M, Muñoz P, Li C, Zhou L, et al (2007) Telomere lengthening early in development. Nat Cell Biol 9:1436–1441. 17982445 10.1038/ncb1664

[12] Olovnikov AM (1973) A theory of marginotomy. The incomplete copying of template margin in enzymic synthesis of polynucleotides and biological significance of the phenomenon. J Theor Biol 41:181–190. 4754905 10.1016/0022-5193(73)90198-7

[13] Harley CB, Futcher AB, Greider CW (1990) Telomeres shorten during ageing of human fibroblasts. Nature 345:458–460. 2342578 10.1038/345458a0

[14] Greider CW (1996) Telomere length regulation. Ann Rev Biochem 65:337–365. 8811183 10.1146/annurev.bi.65.070196.002005

[15] Harley CB, Vaziri H, Counter CM, Allsopp RC (1992) The telomere hypothesis of cellular aging. Exp Gerontol 27:375–382. 1459213 10.1016/0531-5565(92)90068-b

[16] de Lange T (1992) Human telomeres are attached to the nuclear matrix. EMBO J 11:717–724. 1537344 10.1002/j.1460-2075.1992.tb05104.x PMC556504

[17] Ludérus MEE, van Steensel B, Chong L, Sibon OC, Cremers FF, de Lange T (1996) Structure, subnuclear distribution, and nuclear matrix association of the mammalian telomeric complex. J Cell Biol 135:867–881. 8922373 10.1083/jcb.135.4.867 PMC2133388

[18] Amrichová J, Lukášová E, Kozubek S, Kozubek M (2003) Nuclear and territorial topography of chromosome telomeres in human lymphocytes. Exp Cell Res 289:11–26. 12941600 10.1016/s0014-4827(03)00208-8

[19] Nimmo ER, Cranston G, Allshire RC (1994) Telomere-associated chromosome breakage in fission yeast results in variegated expression of adjacent genes. EMBO J 13:3801–3811. 8070408 10.1002/j.1460-2075.1994.tb06691.x PMC395293

[20] Baur JA, Zou Y, Shay JW, Wright WE (2001) Telomere position effect in human cells. Science 292:2075–2077. 11408657 10.1126/science.1062329

[21] Ruiz-Herrera A, Nergadze SG, Santagostino M, Giulotto E (2008) Telomeric repeats far from the ends: mechanisms of origin and role in evolution. Cytogenet Genome Res 122:219–228. 10.1159/000167807 19188690

[22] Wiley JE, Meyne J, Little ML, Stout JC (1992) Interstitial hybridization sites of the (TTAGGG)_n_ telomeric sequence on the chromosomes of some North American hylid frogs. Cytogenet Cell Genet 61:55–57. 1505232 10.1159/000133368

[23] Ocalewicz K (2013) Telomeres in fishes. Cytogenet Genome Res 141:114–125. 10.1159/000354278 23988378

[24] Nanda I, Schrama D, Feichtinger W, Haaf T, Schartl M, Schmid M (2002) Distribution of telomeric (TTAGGG)_n_ sequences in avian chromosomes. Chromosoma 111:215–227. 12424522 10.1007/s00412-002-0206-4

[25] Ventura K, Silva MJ, Fagundes V, Christoff AU, Yonenaga-Yassuda Y (2006) Non-telomeric sites as evidence of chromosomal rearrangement and repetitive (TTAGGG)_n_ arrays in heterochromatic and euchromatic regions in four species of *Akodon* (Rodentia, Muridae). Cytogenet Genome Res 115:169–175. 17065799 10.1159/000095238

[26] Rovatsos MT, Marchal JA, Romero-Fernández I, Fernández FJ, Giagia-Athanosopoulou EB, Sánchez A (2011) Rapid, independent, and extensive amplification of telomeric repeats in pericentromeric regions in karyotypes of arvicoline rodents. Chromosome Res 19:869–882. 10.1007/s10577-011-9242-3 21979796

[27] Nagamachi CY, Pieczarka JC, O'Brien PC, Pinto JA, Malcher SM, Pereira AL, et al (2013) FISH with whole chromosome and telomeric probes demonstrates huge karyotypic reorganization with ITS between two species of *Oryzomyini* (Sigmodontinae, Rodentia): Hylaeamys megacephalus probes on Cerradomys langguthi karyotype. Chromosome Res 21:107–119. 10.1007/s10577-013-9341-4 23494775

[28] Metcalfe CJ, Eldridge MD, Toder R, Johnston PG (1998) Mapping the distribution of the telomeric sequence (T_2_AG_3_)_n_ in the Macropodoidea (Marsupialia), by fluorescence *in situ* hybridization. I. The swamp wallaby, *Wallabia bicolor* . Chromosome Res 6:603–610. 10099873 10.1023/a:1009249325574

[29] Metcalfe CJ, Eldridge MD, Johnston PG (2002) Mapping the distribution of the telomeric sequence (T_2_AG_3_)_n_ in rock wallabies, *Petrogale* (Marsupialia: Macropodidae), by fluorescence *in situ* hybridization. II. The lateralis complex. Cytogenet Genome Res 96:169–175. 12438794 10.1159/000063037

[30] Metcalfe CJ, Eldridge MD, Johnston PG (2007) Mapping the distribution of the telomeric sequence (T_2_AG_3_)_n_ in the Macropodoidea (Marsupialia) by fluorescence *in situ* hybridization. II. The ancestral 2n = 22 macropodid karyotype. Cytogenet Genome Res 116:212–217. 17317962 10.1159/000098189

[31] Garagna S, Ronchetti E, Mascheretti S, Crovella S, Formenti D, Rumpler Y, et al (1997) Non-telomeric chromosome localization of (TTAGGG)_n_ repeats in the genus *Eulemur* . Chromosome Res 5:487–491. 9421267 10.1023/a:1018425215516

[32] Lin KW, Yan J (2008) Endings in the middle: current knowledge of interstitial telomeric sequences. Mutat Res 658:95–110. 17921045 10.1016/j.mrrev.2007.08.006

[33] Hastie ND, Allshire RC (1989) Human telomeres: fusion and interstitial sites. Trends Genet 5:326–331. 2692239 10.1016/0168-9525(89)90137-6

[34] Farré M, Ponsà M, Bosch M (2009) Interstitial telomeric sequences (ITSs) are not located at the exact evolutionary breakpoints in primates. Cytogenet Genome Res 124:128–131. 10.1159/000207517 19420924

[35] Nergadze SG, Rocchi M, Azzalin CM, Mondello C, Giulotto E (2004) Insertion of telomeric repeats at intrachromosomal break sites during primate evolution. Genome Res 14:1704–1710. 15310657 10.1101/gr.2778904 PMC515315

[36] Nergadze SG, Santagostino MA, Salzano A, Mondello C, Giulotto E (2007) Contribution of telomerase RNA retrotranscription to DNA double-strand break repair during mammalian genome evolution. Genome Biol 8:R260 18067655 10.1186/gb-2007-8-12-r260 PMC2246262

[37] Azzalin CM, Nergadze SG, Giulotto E (2001) Human intrachromosomal telomeric-like repeats: sequence organization and mechanisms of origin. Chromosoma 110:75–82. 11453557 10.1007/s004120100135

[38] Lee C, Sasi R, Lin CC (1993) Interstitial localization of telomeric DNA sequences in the Indian muntjac chromosomes: further evidence for tandem chromosome fusions in the karyotypic evolution of the Asian muntjacs. Cytogenet Cell Genet 63:156–159. 8485991 10.1159/000133525

[39] Slijepcevic P (1998) Telomeres and mechanisms of Robertsonian fusion. Chromosoma 107:136–140. 9601982 10.1007/s004120050289

[40] Schmid M, Feichtinger W, Nanda I, Schakowski R, Visbal Garcia R, Manzanilla Puppo J, et al (1994) An extraordinarily low diploid chromosome number in the reptile *Gonatodes taniae* (Squamata, Gekkonidae). J Hered 85:255–260. 7930497 10.1093/oxfordjournals.jhered.a111452

[41] Schmid M, Steinlein C, Feichtinger W, Haaf T, Mijares-Urrutia A, Schargel WE, et al (2014) Cytogenetic studies on *Gonatodes* (Reptilia, Squamata, Sphaerodactylidae). Cytogenet Genome Res 21:47–61. 10.1159/000367929 25341844

[42] Pellegrino KCM, Rodrigues MT, Yonenaga-Yassuda Y (1999) Chromosomal evolution in the Brazilian lizards of genus *Leposoma* (Squamata, Gymnophthalmidae) from Amazon and Atlantic rain forests: banding patterns and FISH of telomeric sequences. Hereditas 131:15–21. 10628293 10.1111/j.1601-5223.1999.00015.x

[43] Pellegrino KCM, dos Santos RML, Rodrigues MT, Laguna MM, Amaro RC, Yonenaga-Yassuda (2009) Chromosomal evolution in the Brazilian geckos of the genus *Gymnodactylus* (Squamata, Phyllodactylidae) from the biomes of Cerrado, Caatinga and Atlantic rain forest: evidence of Robertsonian fusion events and supernumerary chromosomes. Cytogenet Genome Res 127:191–203. 10.1159/000295175 20215729

[44] Bertolotto CEV, Rodrigues MT, Yonenaga-Yassuda Y (2001) Banding patterns, multiple sex chromosome system and localization of telomeric (TTAGGG)_n_ sequences by FISH on two species of *Polychrus* (Squamata, Polychrotidae). Caryologia 54:217–226.

[45] Srikulnath K, Matsubara K, Uno Y, Thongpan A, Suputtitada S, Apisitwanich S, et al (2009) Karyological characterization of the butterfly lizard (*Leiolepis reevesii rubritaeniata*, Agamidae, Squamata) by molecular cytogenetic approach. Cytogenet Genome Res 125:213–223. 10.1159/000230005 19738381

[46] Srikulnath K, Uno Y, Matsubara K, Thongpan A, Suputtitada S, Apisitwanich S, et al (2011) Chromosomal localization of the 18S-28S and 5S rRNA genes and (TTAGGG)_n_ sequences of butterfly lizards (*Leiolepis belliana belliana* and *Leiolepis boehmei*, Agamidae, Squamata). Genet Mol Biol 586:582–586. 10.1590/S1415-47572011005000042 PMC3229112 22215961

[47] Lutes AA, Neaves WB, Baumann DP, Wiegraebe W, Baumann P (2010) Sister chromosome pairing maintains heterozygosity in parthenogenetic lizards. Nature 464:283–286. 10.1038/nature08818 20173738 PMC2840635

[48] Gornung E, Mosconi F, Annesi F, Castiglia R (2013) The first cytogenetic description of *Euleptes europaea* (Gené, 1839) from Northern Sardinia reveals the highest diploid chromosome number among sphaerodactylid geckos (Sphaerodactylidae, Squamata). Comp Cytogenet 7:153–161. 10.3897/CompCytogen.v7i2.4881 24260697 PMC3833756

[49] Young MJ, O'Meally D, Sarre SD, Georges A, Ezaz T (2013) Molecular cytogenetic map of the central bearded dragon, *Pogona vitticeps* (Squamata: Agamidae). Chromosome Res 21:361–374. 10.1007/s10577-013-9362-z 23703235

[50] Johnson Pokorná M, Rovatsos M, Kratochvíl L (2014) Sex chromosomes and karyotype of the (nearly) mythical creature, the Gila monster, *Heloderma suspectum* (Squamata: Helodermatidae). PLoS ONE 9:e104716 10.1371/journal.pone.0104716 25119263 PMC4131918

[51] Koubová M, Johnson Pokorná M, Rovatsos M, Farkačová K, Altmanová M, Kratochvíl L (2014) Sex determination in Madagascar geckos of the genus *Paroedura* (Squamata: Gekkonidae): are differentiated sex chromosomes indeed so evolutionary stable? Chromosome Res 22:441–452. 10.1007/s10577-014-9430-z 25056523

[52] Pokorná M, Rens W, Rovatsos M, Kratochvíl L (2014) A ZZ/ZW sex chromosome system in the thick-tailed gecko (*Underwoodisaurus milii*; Squamata: Gekkota: Carphodactylidae), a member of the ancient gecko lineage. Cytogenet Genome Res 142:190–196. 10.1159/000358847 24603160

[53] Rojo V, Giovannotti M, Naveira H, Nisi Cerioni P, González-Tizón AM, Caputo Barucchi V, et al (2014) Karyological characterization of the endemic Iberian rock lizard, *Iberolacerta monticola* (Squamata, Lacertidae): insights into sex chromosome evolution. Cytogenet Genome Res 142:28–39. 10.1159/000356049 24296524

[54] Matthey R (1931) Chromosomes des reptiles, sauriens, ophidiens, chéloniens. L’évolution de la formule chromosomiale chez les sauriens. Rev Suisse Zool 38:117–186.

[55] Gorman GC (1973) The chromosomes of the Reptilia, a cytotaxonomic interpretation In: Chiarelli AB, Capanna E, editors. Cytotaxonomy and Vertebrate Evolution. New York: Academic Press pp. 349–424.

[56] King M (1991) Chromosome change and speciation in lizards In: Atchley WR, Woodruff DS, editors. Evolution and Speciation. Cambridge: Cambridge University Press pp. 262–285.

[57] Giovannotti M, Caputo V, O'Brien PC, Lovell FL, Trifonov V, Cerioni PN, et al (2009) Skinks (Reptilia: Scincidae) have highly conserved karyotypes as revealed by chromosome painting. Cytogenet Genome Res 127:224–231. 10.1159/000295002 20215726

[58] Pokorná M, Giovannotti M, Kratochvíl L, Kasai F, Trifonov VA, O'Brien PC, et al (2011) Strong conservation of the bird Z chromosome in reptilian genomes is revealed by comparative painting despite 275 million years divergence. Chromosoma 120:455–468. 10.1007/s00412-011-0322-0 21725690

[59] Pokorná M, Giovannotti M, Kratochvíl L, Caputo V, Olmo E, Ferguson-Smith MA, et al (2012) Conservation of chromosomes syntenic with avian autosomes in squamate reptiles revealed by comparative chromosome painting. Chromosoma 121:409–418. 10.1007/s00412-012-0371-z 22619043

[60] Trifonov VA, Giovannotti M, O'Brien PCM, Wallduck M, Lovell F, Rens W, et al (2011) Chromosomal evolution in Gekkonidae. I. Chromosome painting between *Gekko* and *Hemidactylus* species reveals phylogenetic relationships within the group. Chromosome Res 19:843–855. 10.1007/s10577-011-9241-4 21987185

[61] Srikulnath K, Nishida C, Matsubara K, Uno Y, Thongpan A, Suputtitada S, et al (2009) Karyotypic evolution in squamate reptiles: comparative gene mapping revealed highly conserved linkage homology between the butterfly lizard (*Leiolepis reevesii rubritaeniata*, Agamidae, Lacertilia) and the Japanese four-striped rat snake (*Elaphe quadrivirgata*, Colubridae, Serpentes). Chromosome Res 17:975–986. 10.1007/s10577-009-9101-7 19937109

[62] Srikulnath K, Uno Y, Nishida C, Matsuda Y (2013) Karyotype evolution in monitor lizards: cross-species chromosome mapping of cDNA reveals highly conserved synteny and gene order in the Toxicofera clade. Chromosome Res 21:805–819. 10.1007/s10577-013-9398-0 24343421

[63] Gamble T, Geneva AJ, Glor RE, Zarkower D (2014) *Anolis* sex chromosomes are derived from a single ancestral pair. Evolution 68:1027–1041. 10.1111/evo.12328 24279795 PMC3975651

[64] Rovatsos M, Altmanová M, Pokorná M, Kratochvíl L (2014) Conserved sex chromosomes across adaptively radiated *Anolis* lizards. Evolution 68:2079–2085. 10.1111/evo.12357 24433436

[65] Rovatsos M, Pokorná M, Altmanová M, Kratochvíl L (2014) Cretaceous park of sex determination: sex chromosomes are conserved across iguanas. Biol Lett 10:20131093 10.1098/rsbl.2013.1093 24598109 PMC3982436

[66] Rovatsos M, Altmanová M, Johnson Pokorná M, Kratochvíl L (2014) Novel X-linked genes revealed by qPCR in the green anole, *Anolis carolinensis* . G3-Genes Genom Genet 4:2107–2113. 10.1534/g3.114.014084 PMC4232536 25172916

[67] Pokorná M, Rábová M, Ráb P, Ferguson-Smith MA, Rens W, Kratochvíl L (2010) Differentiation of sex chromosomes and karyotypic evolution in the eye-lid geckos (Squamata: Gekkota: Eublepharidae), a group with different modes of sex determination. Chromosome Res 18:809–820. 10.1007/s10577-010-9154-7 20811940

[68] Ijdo JW, Wells RA, Baldini A, Reeders ST (1991) Improved telomere detection using a telomere repeat probe (TTAGGG)_n_ generated by PCR. Nucleic Acids Res 19:4780 1891373 10.1093/nar/19.17.4780 PMC328734

[69] Maddison WP, Maddison DR (2011) Mesquite: a modular system for evolutionary analysis. Version 2.75. http://mesquiteproject.org

[70] Pyron RA, Burbrink FT, Wiens JJ (2013) A phylogeny and revised classification of Squamata, including 4161 species of lizards and snakes. BMC Evol Biol 13:93 10.1186/1471-2148-13-93 23627680 PMC3682911

[71] Rovatsos MT, Marchal A, Romero-Fernández I, Cano-Linares M, Fernández FJ, Giagia-Athanasopoulou EB, et al (2014) Molecular and physical characterization of the complex pericentromeric heterochromatin of the vole species *Microtus thomasi* . Cytogenet Genome Res 144:131–141. 10.1159/000368648 25402553

[72] Völker M, Backström N, Skinner BM, Langley EJ, Bunzey SK, Ellegren H, et al (2010) Copy number variation, chromosome rearrangement, and their association with recombination during avian evolution. Genome Res 20:503–511. 10.1101/gr.103663.109 20357050 PMC2847753

[73] Skinner BM, Griffin DK (2012) Intrachromosomal rearrangements in avian genome evolution: evidence for regions prone to breakpoints. Heredity 108:37–41. 10.1038/hdy.2011.99 22045382 PMC3238122

[74] Lithgow PE, O'Connor R, Smith D, Fonseka G, Al Mutery A, Rathje C, et al (2014) Novel tools for characterising inter and intra chromosomal rearrangements in avian microchromosomes. Chromosome Res 22:85–97. 10.1007/s10577-014-9412-1 24696127

[75] Alföldi J, Di Palma F, Grabherr M, Williams C, Kong L, Mauceli E, et al (2011) The genome of the green anole lizard and a comparative analysis with birds and mammals. Nature 477:587–591. 10.1038/nature10390 21881562 PMC3184186

[76] Johnson Pokorná M, Trifonov VA, Rens W, Ferguson-Smith MA, Kratochvíl L (2015) Low rate of interchromosomal rearrangements during old radiation of gekkotan lizards (Squamata: Gekkota). Chromosome Res, in press. 10.1007/s10577-015-9468-6 25665924

[77] Ezaz T, Deakin JE (2014) Repetitive sequence and sex chromosome evolution in vertebrates. Adv Evol Biol 2014:1–9.

[78] Gomes NMV, Shay JW, Wright WE (2010) Telomere Biology in Metazoa. FEBS Lett 584:3741–3751. 10.1016/j.febslet.2010.07.031 20655915 PMC2928394

[79] Christiansen JL, Johnson JC, Henderson ER, Budke BJ, Lynch M (2001) The relationship between telomeres, telomerase, reptilian lifespan, and reptilian tissue regeneration. Proceedings of the Iowa Space Grant Consortium 2001:1–10.

[80] Ujvari B, Madsen T (2009) Short telomeres in hatchling snakes: erythrocyte telomere dynamics and longevity in tropical pythons. PLoS ONE 4:e7493 10.1371/journal.pone.0007493 19834611 PMC2759514

[81] Laguna MM, Amaro RC, Mott T, Yonenaga-Yassuda Y, Rodrigues MT (2010) Karyological study of *Amphisbaena ridleyi* (Squamata, Amphisbaenidae), an endemic species of the Archipelago of Fernando de Noronha, Pernambuco, Brazil. Genet Mol Biol 33:57–61. 10.1590/S1415-47572010005000009 21637605 PMC3036086

